# Why digitally-enabled health system transformation needs different forms of innovation

**DOI:** 10.1136/bmjhci-2020-100173

**Published:** 2020-07-22

**Authors:** Kathrin Cresswell, Robin Williams, Narath Carlile, Aziz Sheikh

**Affiliations:** 1 Usher Institute, The University of Edinburgh, Edinburgh, UK; 2 Institute for the Study of Science, Technology and Innovation, The University of Edinburgh, Edinburgh, UK; 3 Brigham and Women’s Hospital, Harvard Medical School, Boston, Massachusetts, USA

**Keywords:** health care, medical informatics

Health systems face major challenges, including an ageing multimorbid population and the need to deliver a greater array of interventions with finite resources.^1^ If health systems are to continue to be able to cater for the populations they serve, they will need organisational, service and social innovation as well as product innovation.

Policymakers, particularly in high-income countries, recognising the need to tackle existing challenges in new ways, are investing in a range of innovation centres (and related initiatives such as sandpits, living labs, hackathons) to try and find new ways of responding to these needs. Most current healthcare innovation centres are, however, focussed on early stage (often digital) product innovation where tools are designed and developed to tackle existing healthcare challenges.^2^ Although important, product innovation in isolation is insufficient to bring about transformative digitally-enabled health system-wide changes. Other forms of innovation need to become integral to healthcare innovation activity and accompany the development of new products. These include organisational, service and social innovation.

**Figure 1:**
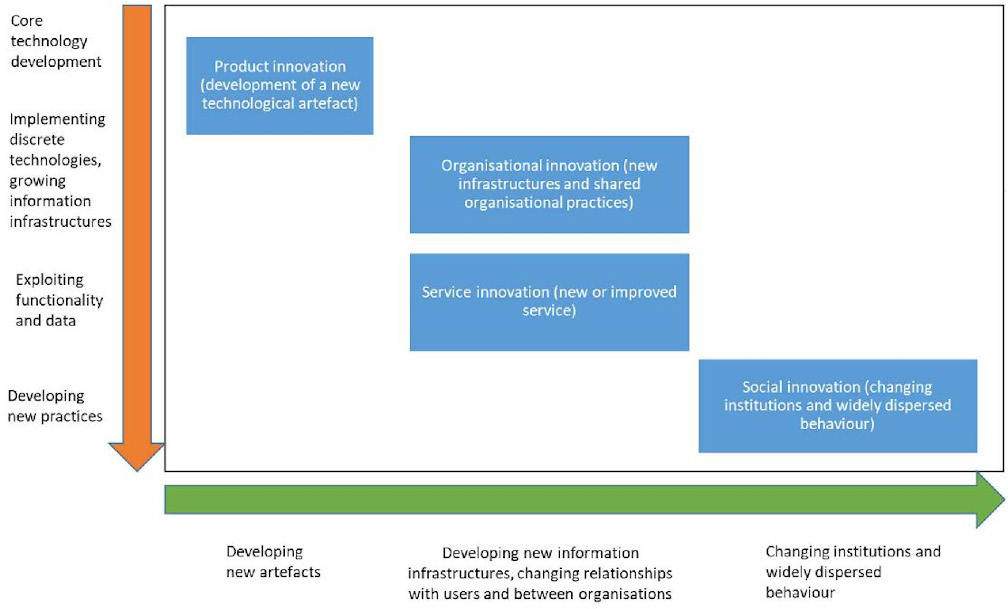
Different forms of innovation required for effective digitally-enabled health system change (adapted from^11^).

Figure 1 highlights how these different forms of innovation may be conceptualised. It shows that digital product innovation is only a small component of the diverse forms of innovation needed to transform how care is delivered across organisational boundaries, to support patients in managing their health, and in preventing disease. The implementation of new technologies can only be truly transformative, with far reaching impacts across a range of stakeholders, if it goes hand-in-hand with organisational, service and social innovation.^3–5^

Organisational innovation is needed to ensure that digital technologies are effectively integrated with healthcare services and existing organisational technologies and bring maximum benefits to patients.^6^ For example, to maximise the opportunities of moving to remote models of care, patient-facing technologies need to be integrated within existing electronic health record platforms and thereby allow healthcare professionals to access extensive and timely information.^7^ However, more data will not automatically deliver benefits as healthcare professionals are already faced with data overload and need to change their work practices to integrate new technologies and new information flows.^8^ As a result, the way services are delivered needs to change (service innovation). If these changes are then also integrated with social innovation, where new social practices are established that meet social needs better than existing solutions, the transformative impact on the health system can be further reinforced. For instance, connecting existing health information infrastructures at scale can enable population risk management and help to establish new transformative health-system models.^9^

Promoting product innovation in healthcare is necessary but insufficient for transforming health systems as it may unhelpfully direct attention towards automation of a limited set of activities at the expense of wider transformation.^10^ There is therefore an urgent need for policymakers to stimulate the emergence of organisational, service, social innovation pathways in addition to product innovation. This may be achieved through creating mechanisms to recognise these other types of innovations to help raise their profile and stimulate organisational and social investment in them.
